# Urinary bladder paraganglioma presenting with abdominal pain and elevated cardiac enzymes: a case report of atypical manifestations and diagnostic challenges

**DOI:** 10.3389/fonc.2025.1651275

**Published:** 2025-12-04

**Authors:** Hong Zhou, Xiaohui Hu, Qin Yan

**Affiliations:** Department of Gynecology, Shanghai First Maternity and Infant Hospital, School of Medicine, Tongji University, Shanghai, China

**Keywords:** urinary bladder paraganglioma, cardiac enzymes, abdominal pain, case report, QTc

## Abstract

Urinary bladder paraganglioma (UBPGL) is a very rare neuroendocrine tumor, accounting for 0.05% of all bladder neoplasms. Classic symptoms include paroxysmal hypertension, palpitations, and sweating triggered by micturition. However, atypical presentations may complicate the diagnosis. This report describes the case of a 35-year-old woman who presented with persistent lower abdominal pain and isolated elevation of cardiac enzymes. Imaging revealed a pelvic mass initially suspected to be adnexal pathology. Emergency laparoscopy identified a retroperitoneal mass adjacent to the bladder and an endometriotic ovarian cyst. Postoperative pathology confirmed UBPGL, with immunohistochemistry positive for neuroendocrine markers (CD56, chromogranin-A, synaptophysin). Elevated cardiac enzymes and a prolonged QTc interval were observed preoperatively, likely secondary to catecholamine-induced myocardial injury, although biochemical confirmation was not available. The patient recovered uneventfully after resection of the tumor. This case highlights the potential for UBPGL to have atypical cardiovascular manifestations, including isolated elevation of cardiac enzymes without classic hypertensive crises. Such findings may reflect catecholamine-driven cardiomyocyte stress, emphasizing the need for heightened suspicion in cases that present with unexplained abdominal pain and cardiac abnormalities. Diagnostic challenges arise from overlapping symptoms with gynecologic emergencies and the rarity of preoperative biochemical testing in acute settings. UBPGL should be considered in patients with a pelvic mass accompanied by unexplained cardiac abnormalities. Early resection remains curative, but multidisciplinary evaluation, including biochemical screening for catecholamine excess, is critical to mitigate the cardiovascular risk. Long-term surveillance is warranted in view of the potential for metastasis. This case underscores the importance of including rare neuroendocrine tumors in the differential diagnoses for atypical abdominal presentations.

## 1 Introduction

Paraganglioma is a rare neuroendocrine tumor originating from extra-adrenal neuroendocrine tissue, with approximately one third to half of cases located in the thoracoabdominal region (1).Urinary bladder paraganglioma (UBPGL), a subtype of paraganglioma, is exceedingly rare, arising from paraganglia within the bladder wall, and constitutes 0.7% of all paragangliomas and 0.05% of all bladder tumors (2). The rarity of UBPGL presents challenges in terms of clinical presentation, diagnosis, and management. Common clinical manifestations include paroxysmal hypertension, headache, palpitations, and sweating, which are typically triggered by micturition or bladder manipulation as a result of intermittent catecholamine release from the tumor (3). However, atypical manifestations can complicate the diagnostic process. This report describes a case of UBPGL that presented with abdominal pain and elevated cardiac enzymes and highlights the atypical clinical presentation, diagnostic challenges, and treatment of paragangliomas.

## 2 Case presentation

**Figure 1:**
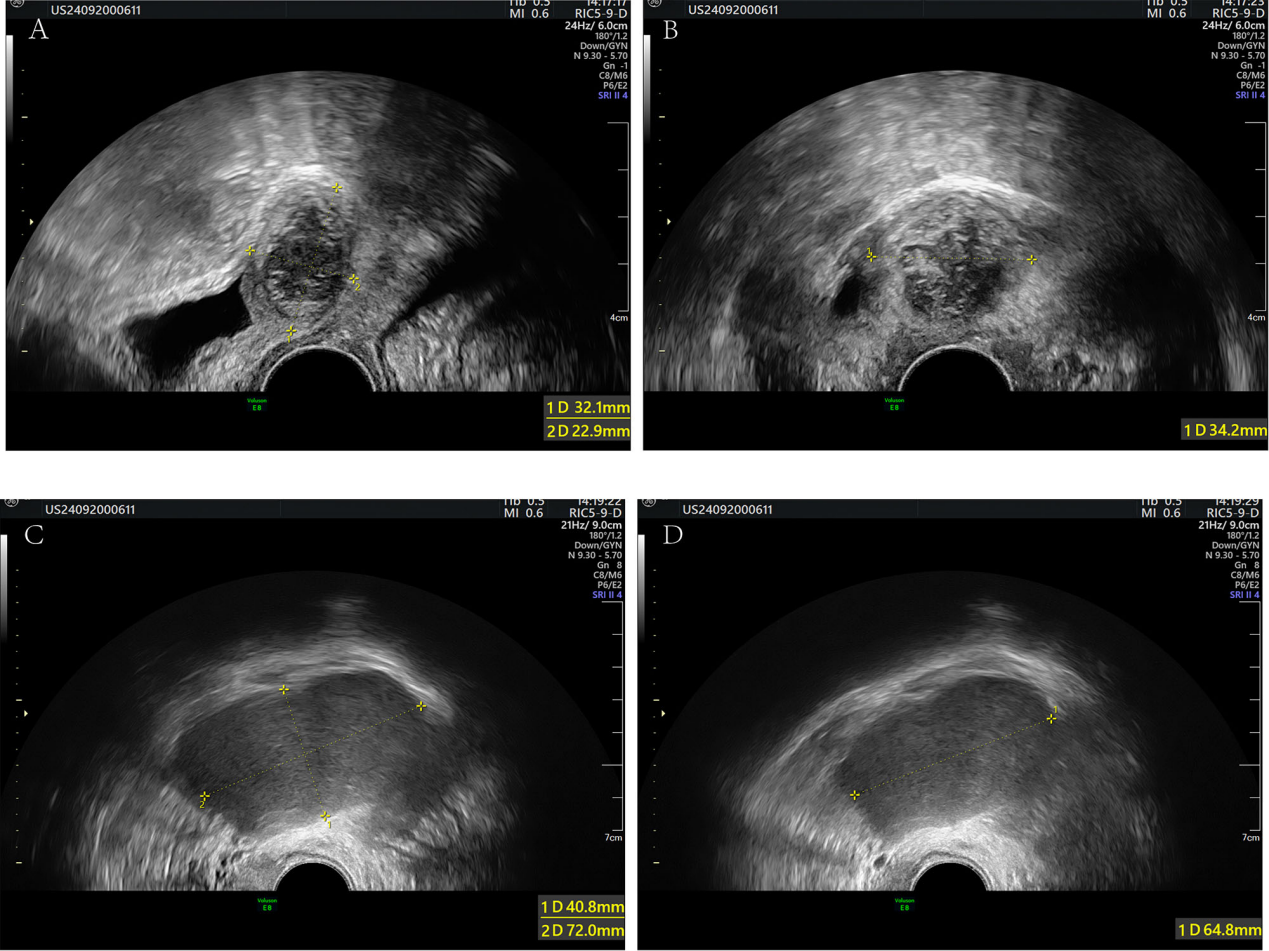
Transvaginal ultrasound showed a hypoechoic structure between the cervix and bladder, measuring 32X34X23mm **(A, B)**. and the right ovary showed hypoechoic areas measuring 42X72X64 mm **(C, D)**.

**Figure 2:**
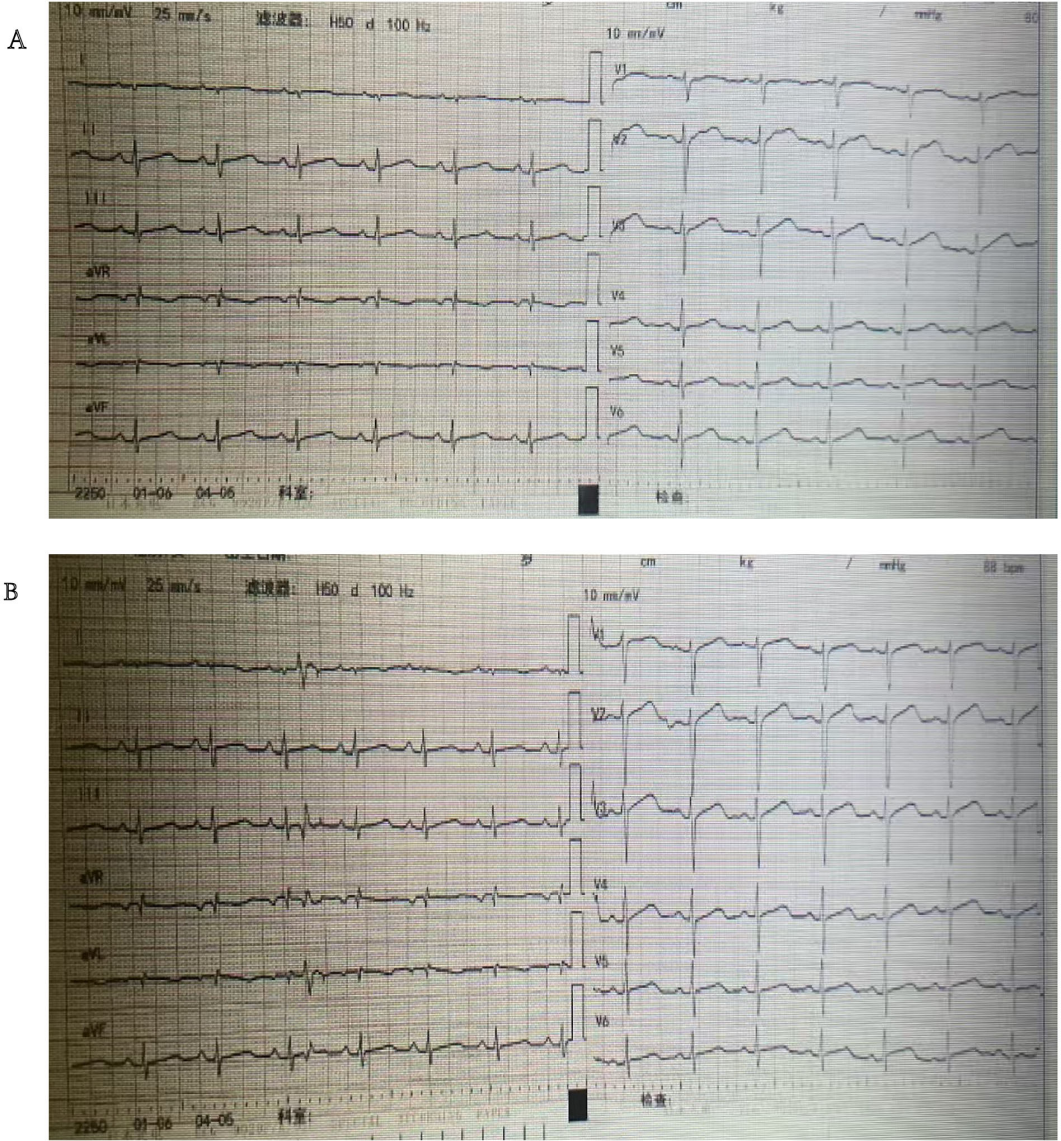
ECG showed prolonged QTc interval before operation and after operation; **(A)** before operation; **(B)** 2 hours after operation.

**Figure 3:**
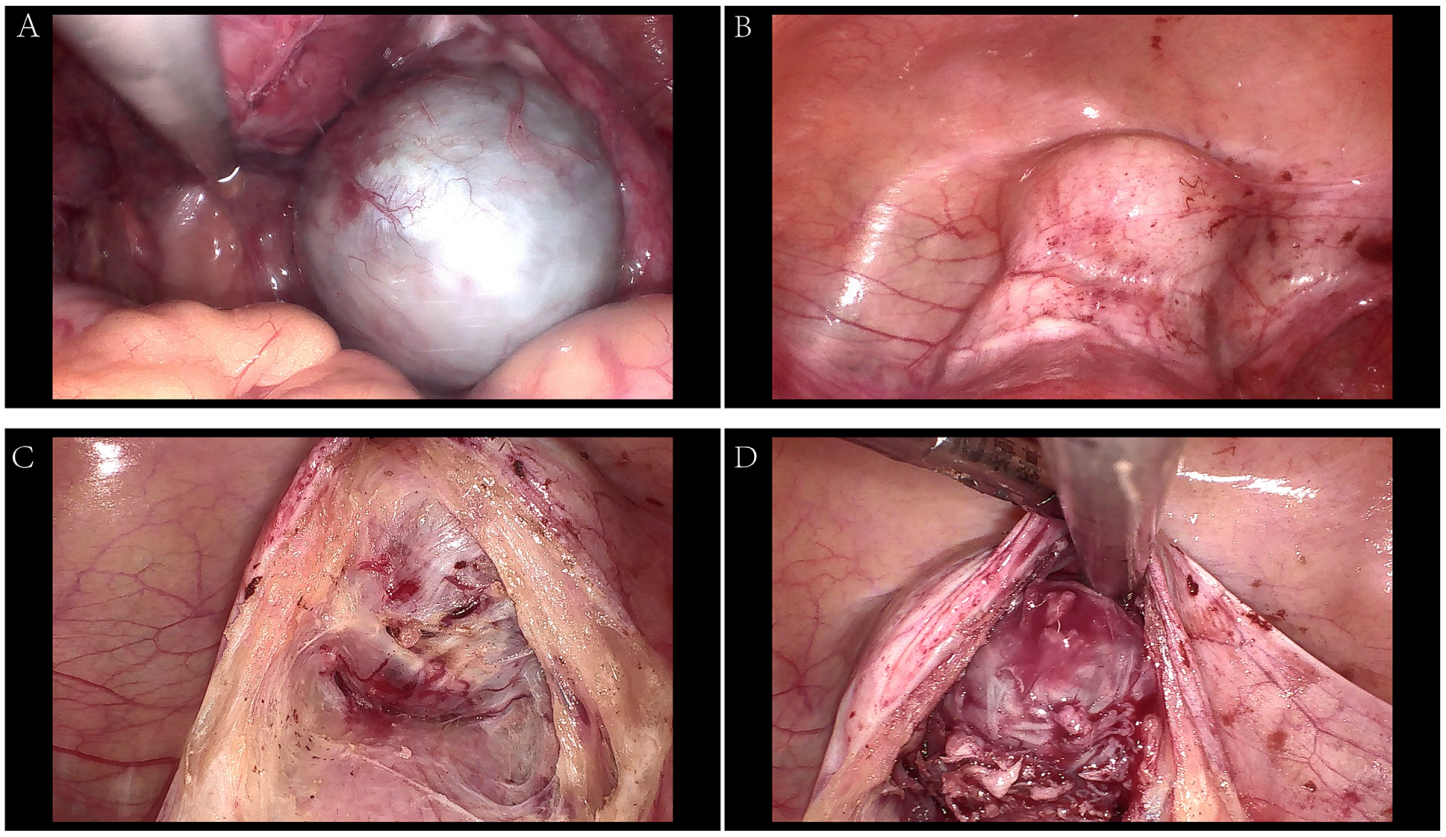
Intraoperative images. **(A)** the right ovary cast; **(B–D)** the bladder Paraganglioma.

The patient was a 35-year-old married nulliparous woman with no history of chronic diseases or surgical procedures who presented with complaints of persistent lower abdominal pain and a sensation of rectal heaviness. Abdominal CT performed at another hospital revealed a low-density mass measuring 42×80 mm in the right pelvic floor space, with increased edge density and clear boundaries. The patient visited our outpatient clinic with worsening abdominal pain. On presentation, she had a temperature of 36.5 °C, a blood pressure of 122/82 mmHg, a heart rate of 71 beats per minute, and an SpO_2_ of 99%. Physical examination revealed significant abdominal tenderness and guarding, with equivocal rebound tenderness. A vaginal examination identified marked cervical tenderness, mild uterine tenderness, and a 6-cm mass in the right adnexal region with unclear boundaries and considerable tenderness. Ultrasonography revealed a hypoechoic area measuring 32×34×23 mm between the cervix and bladder, with clear boundaries, poor transmission, short linear hyperechoic structures inside, and sparse blood flow signals on the capsule; the right ovary showed a hypoechoic area measuring 42×72×64 mm (**Figure 1**). Considering adnexal torsion or rupture as a potential cause of the patient’s abdominal pain, she was admitted for emergency surgery. Her routine blood tests, coagulation profile, electrolytes level, liver and kidney function tests were normal on admission, and an electrocardiogram showed a heart rate of 80 beats per minute with a prolonged QTc interval and no ST-T segment changes (**Figure 2**). An emergency laparoscopy was performed, with a preoperative blood pressure of 145/95 mmHg, a heart rate of 90 beats per minute, and an SpO_2_ of 99%. Intraoperatively, the right ovary was found to be enlarged, measuring 7 cm in diameter, and to contain a cyst filled with chocolate-like fluid. There were no signs of cyst rupture or torsion of the ovary, and the cyst was excised (**Figure 3A**). A 4×3×2-cm mass behind the bladder and in front of the uterus was also identified and excised (**Figures 3B–D**). Both specimens were sent for pathological examination.

The intraoperative laboratory report indicated that the cardiac enzyme profile had been abnormally elevated preoperatively, showed as followed: brain natriuretic peptide (BNP)694 pg/ml (reference range<100 pg/ml), CTnI 3.918ng/ml ((reference range<0.05ng/ml) and Creatine Kinase MB (CKMB)31 ng/ml (reference range <0.5ng/ml) (**Table 1**). Immediately after surgery, rechecking of the cardiac enzyme profile showed that it was still elevated (**Table 1**), and the electrocardiogram again showed a prolonged QTc interval with no other abnormalities. Bedside echocardiography revealed an ejection fraction of 64%, normal cardiac chamber sizes, no organic changes in the valves, normal thickness and amplitude of the interventricular septum and left ventricular posterior wall, and mild tricuspid regurgitation. A CT scan showed scattered flocculent shadows in both lungs, with no other significant abnormalities. The patient was transferred to the intensive care unit for observation and treatment, and subsequent follow-up showed a gradual decrease in cardiac enzymes to the normal range. The patient recovered well, with a stable temperature, a heart rate of 65–80 beats per minute, and blood pressure maintained at 58–70/90–115 mmHg. There were no complaints of discomfort, and she was discharged on the sixth postoperative day. **Table 1** summarizes the patient’s significant laboratory results.

**Table 1:** Significant laboratory results and progress trends.

Summary of the patient's significant laboratory results	Reference range	Before-op	Hour 1	Hour 6	Day1	Day2	Day4	Day6
WBC (10^9/L)	3.5-9.5	14.5	13.07	14.19	7.90	6.66	5.39	4.97
HB (g/L)	115-150	114	99	91	79	76	78	90
PLT (10^9/L)	125-350	491	387	334	244	247	282	354
D-dimer (mg/L)	<0.55	3.42	3.14	3.80	1.29	1.2	1.13	1.48
BNP (pg/ml)	<100	694	762	945	349	90.2	119	75.4
CTnI (ng/ml)	<0.05	3.918	3.355	2.083	0.96	0.42	0.07	<0.05
CKMB (ng/ml)	<5	41	33.3	20.5	4.4	<1	<1	<1

WBC, white blood cell; HB, hemoglobin; PLT, platelet; BNP, brain natriuretic peptide; CTnl, cardiac troponin1; CKMB, Creatine Kinase MB.

**Figure 4:**
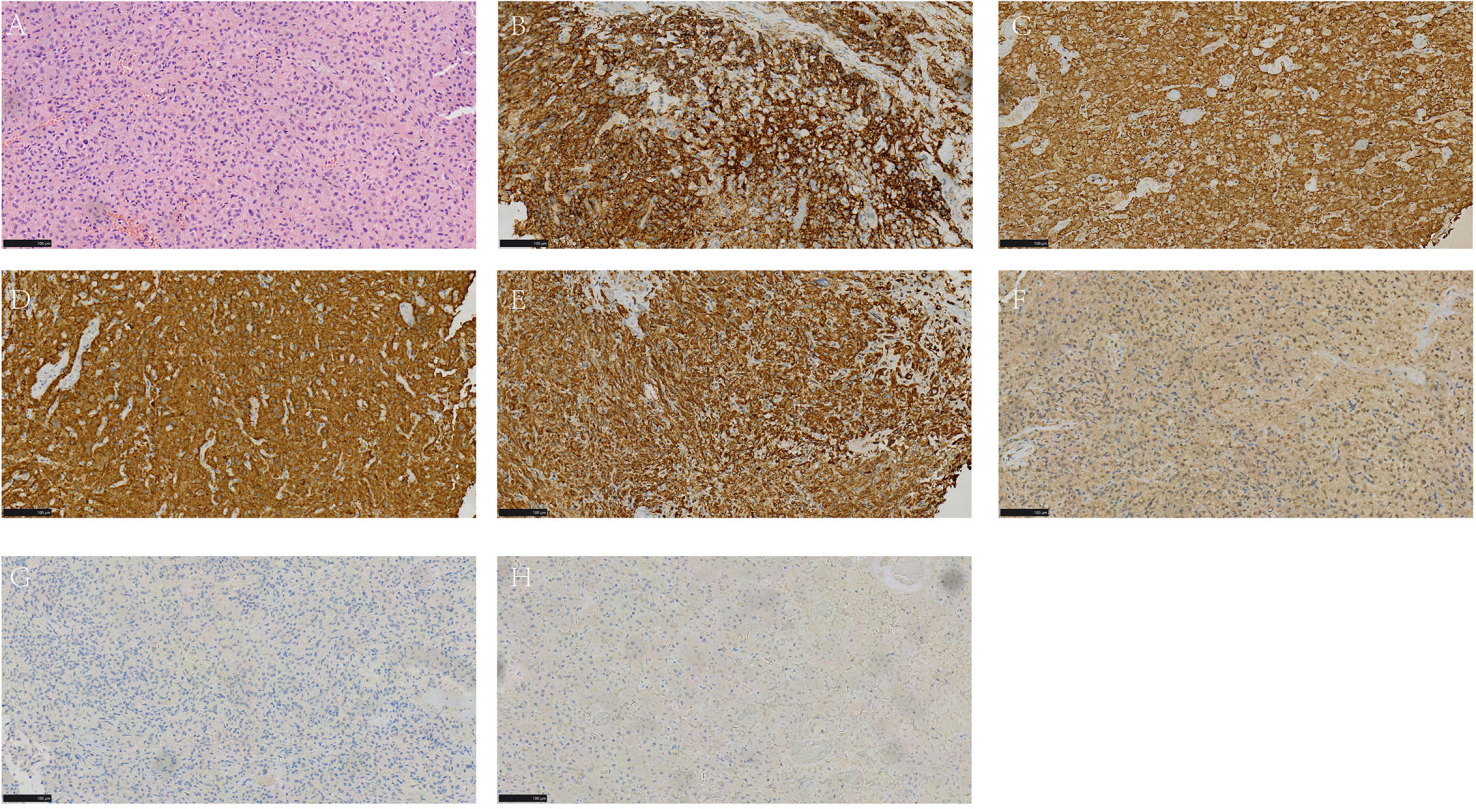
HE and Immunohistochemical staining of tumor cells. **(A)** 200x magnification of HE staining showed Zellballen pattern; **(B–E)** Diffuse and strongly positive for CD56 **(B)**, Chromogranin-A **(C)**, Synaptophysin **(D)**, Vimentin **(E)** Immunohistochemistry; **(F)** less positive for S100 Immunohistochemistry; **(G, H)** negative for CKpan **(G)** and EMA **(H)** Immunohistochemistry. HE, Hematoxylin and Eosin.

The postoperative pathological diagnosis was retroperitoneal paraganglioma of the bladder and endometrial cyst in the right ovary. Hematoxylin–eosin staining of the bladder paraganglioma showed characteristic nests of cells (known as the Zellballen pattern) with eosinophilic cytoplasm and round nuclei. CD56, chromogranin-A, synaptophysin, and vimentin staining was strongly positive. In contrast, S100 staining was positive in patches, and staining for pancytokeratin and epithelial membrane antigen was negative (**Figure 4**).

## 3 Discussion

This case highlights the atypical clinical manifestations and diagnostic challenges associated with paragangliomas. Although our patient had a concurrent endometriotic ovarian cyst, intraoperative laparoscopic examination did not reveal any signs of rupture or torsion. Therefore, the patient’s abdominal pain was unlikely to be related to the cyst. Her abdominal pain was eventually linked to a paraganglioma, and the preoperative elevation of cardiac enzymes may have been related to the cardiac effects of catecholamines secreted by the tumor. However, this case was treated as a surgical emergency, so paraganglioma was not considered preoperatively, and the patient had been discharged by the time the pathological diagnosis was confirmed. And during the subsequent follow-up, the patient refused to undergo genetic testing.

Paraganglioma is a rare tumor originating from cells of the neural crest. Its most common clinical manifestations include paroxysmal hypertension, headaches, palpitations, and sweating, usually triggered by urination or manipulation of the bladder (4). These symptoms are attributed to the intermittent release of catecholamines by the tumor. However, atypical presentations may occur, further complicating the diagnostic process. Patients rarely present with cardiac complications initially. Paraganglioma-induced cardiomyopathy occurs in up to 11% of cases; it is commonly associated with adrenal pheochromocytoma (90%) and rarely with paragangliomas originating from the sympathetic ganglia (10%). The cardiovascular complications of paraganglioma are diverse, and paraganglioma-related cardiomyopathy can be chronic or acute, with Takotsubo cardiomyopathy being the most frequently reported. Presentations with abnormal myocardial enzyme levels alone are rare (5, 6). Moreover, the infrequent occurrence of atypical preoperative presentations poses a significant diagnostic dilemma for urologists (1, 7).

In conclusion, early diagnosis and treatment of paraganglioma is essential, given the potential for this tumor to result in severe cardiovascular complications. Correct diagnosis and treatment can significantly improve the prognosis. This case report underscores the importance of careful evaluation of patients with abdominal pain and elevation of cardiac enzymes, as well as the need to consider rare conditions, such as paraganglioma, in the diagnostic process. Physicians need to be vigilant, maintaining a high index of clinical suspicion for prompt and effective treatment, even in cases of patient-reported symptoms or atypical differential diagnoses (or rare coexistences). Even in cases of negative lab and imaging workup, a small lesion can be hidden in the bladder. Surgical resection remains the only effective treatment for paraganglioma, involving partial or complete cystectomy, some reports recommended transurethral resection of bladder tumor (TURBT) (8, 9). Long-term clinical follow-up include genetic background analysis such as SDHB, SDHD, VHL, and RET, required in view of the metastatic potential of this rare neuroendocrine tumor.

## Data Availability

The original contributions presented in the study are included in the article/supplementary material. Further inquiries can be directed to the corresponding author.

## References

[1] Symeonidis EN Symeonidis A Gkekas C Georgiadis C Malioris A Papathanasiou M . Urothelial neoplasm in a 19-year-old male patient with urine discoloration, negative lab, and imaging workup: Should we investigate the findings or the symptom? Clin Case Rep. (2019) 7:409–12. doi: 10.1002/ccr3.1909, PMID: 30899460 PMC6406157

[2] Kouba E Cheng L . Neuroendocrine tumors of the urinary bladder according to the 2016 world health organization classification: molecular and clinical characteristics. Endocr Pathol. (2016) 27:188–99. doi: 10.1007/s12022-016-9444-5, PMID: 27334654

[3] Yu K Ebbehoj AL Obeid H Vaidya A Else T Wachtel H . Presentation, management, and outcomes of urinary bladder paraganglioma: results from a multicenter study. J Clin Endocrinol Metab. (2022) 107:2811–21. doi: 10.1210/clinem/dgac427, PMID: 35882219 PMC9516048

[4] Mete O Asa SL Gill AJ Kimura N de Krijger RR Tischler A . Overview of the 2022 WHO classification of paragangliomas and pheochromocytomas. Endocr Pathol. (2022) 33:90–114. doi: 10.1007/s12022-022-09704-6, PMID: 35285002

[5] Petrak O Kratka Z Holaj R Zitek M Nguyen Nikrynova T Klimova J . Cardiovascular complications in pheochromocytoma and paraganglioma: does phenotype matter? Hypertension. (2024) 81:595–603. doi: 10.1161/HYPERTENSIONAHA.123.21902, PMID: 38152977

[6] Szatko A Glinicki P Gietka-Czernel M . Pheochromocytoma/paraganglioma-associated cardiomyopathy. Front Endocrinol (Lausanne). (2023) 14:1204851. doi: 10.3389/fendo.2023.1204851, PMID: 37522121 PMC10374018

[7] Symeonidis A Tsikopoulos I Symeonidis EN Tsifountoudis I Michailidis A Tsantila I . More than meets the eye: A case of synchronous ipsilateral clear cell renal cell carcinoma and urothelial carcinoma of the pelvicalyceal system and literature review. Acta Biomed. (2022) 92:e2021380. doi: 10.23750/abm.v92i6.11768, PMID: 35075075 PMC8823562

[8] Symeonidis EN Lo KL Chui KL Vakalopoulos I Sountoulides P . En bloc resection of bladder tumors: challenges and unmet needs in 2022. Future Oncol. (2022) 18:2545–58. doi: 10.2217/fon-2021-1228, PMID: 35642479

[9] Matsumoto S Ishikawa Y Fukushima H Yamamoto K Tsujimoto K Kimura K . A case of bladder paraganglioma completely resected by transurethral endoscopic en-bloc resection of bladder tumor. IJU Case Rep. (2025) 8:93–6. doi: 10.1002/iju5.12811, PMID: 40034905 PMC11872215

